# Circular RNAs in cancer: new insights into functions and implications in ovarian cancer

**DOI:** 10.1186/s13048-019-0558-5

**Published:** 2019-09-03

**Authors:** Zahra Shabaninejad, Asma Vafadar, Ahmad Movahedpour, Younes Ghasemi, Afshin Namdar, Hadis Fathizadeh, Mohammad Hossein Pourhanifeh, Amir Savardashtaki, Hamed Mirzaei

**Affiliations:** 1 Department of Nanobiotechnology, School of Basic Sciences, Tarbiat Modares University, Tehran, Iran; 2 Pharmaceutical Sciences Research Center, Shiraz University of Medical Sciences, Shiraz, Iran; 3 Department of Medical Biotechnology, School of Advanced Medical Sciences and Technologies, Shiraz University of Medical Sciences, Shiraz, Iran; 4 Student Research Committee, Shiraz University of Medical Sciences, Shiraz, Iran; 5 Department of Pharmaceutical Biotechnology, School of Pharmacy and Pharmaceutical Sciences Research Center, Shiraz University of Medical Sciences, Shiraz, Iran; 6 Department of Dentistry, Faculty of Medicine and Dentistry, University of Alberta, Edmonton, Canada; 7 Department of Microbiology and Immunology, Faculty of Medicine, Kashan University of Medical Sciences, Kashan, Iran; 8 Research Center for Biochemistry and Nutrition in Metabolic Diseases, Institute for Basic Sciences, Kashan University of Medical Sciences, Kashan, Iran

**Keywords:** Circular RNA, MicroRNA, Ovarian cancer

## Abstract

Circular RNAs (circRNAs) are a class of long non-coding RNAs (lncRNAs) which have a circular and closed loop structure. They are ubiquitous, stable, conserved and diverse RNA molecules with a range of activities such as translation and splicing regulation, which are able to interacting with RNA-binding proteins and specially miRNA sponge. The expression patterns of the circRNAs exhibited tissue specificity and also, step and stage specificity. Accumulating evidences approved the critical role of circular RNAs in many cancers such as ovarian cancer. Given that these molecules exert their effects through multiple cellular and molecular mechanisms (i.e., angiogenesis, apoptosis, growth, and metastasis) which are involved in cancer pathogenesis, circular RNAs, in particular, act by controlling cell proliferation in ovarian cancer, so that, it has been shown that the deregulation of these molecules is associated with initiation and progression of ovarian cancer. Therefore, they are attractive molecules which have introduced them as cancer biomarkers. Moreover, they could be used as new therapeutic candidates for developing novel treatment strategies. Here, for first time, we have provided a comprehensive review on the recent knowledge of circular RNAs and their pathological roles in the ovarian cancer.

## Introduction

The majority portion of genome (approximately 98%) is assigned to non-coding RNAs (ncRNAs) rather than protein coding mRNAs [1]. They are categorized into two main group: (1) housekeeper ncRNAs, which are consist of tRNAs, rRNAs, snoRNAs and snRNAs; (2) regulatory ncRNAs. Based on the nucleotide fragment length, the regulatory ncRNAs can be classified into < 200 nucleotides in transcript length, some of which are microRNAs (miRNAs) and siRNAs, and the long non coding RNAs (lncRNAs) which possess transcripts with more than 200 nucleotides in length [2, 3].

Circular RNAs (circRNAs) group is a member of lncRNAs with a large variation in length ranged from hundreds to thousands nucleotides [4]. During the pre-mRNA splicing, the 5′ end of upstream exon and the 3′ end of a downstream exon or the ends of an individual exon could join together to generate a circRNAs. The Circular essence of circRNAs make them more stable against RNase in comparison with linear RNA forms [5]. While majority of circRNAs are originated from coding pre-mRNA, but they are classified as lncRNAs. The circRNAs was firstly discovered in 1979 [6], but the technical limitation hindered the wide identifications and investigations. Until last two decade, emerging rapid advanced in molecular techniques like next generation sequencing, the number of identified circRNAs and researches on their function [7, 8].

Like other lncRNAs, circRNAs could be implicated in RNA or protein sponging to modulate gene expression. The most well-known mechanisms is in miRNA regulation by acting as competing endogenous miRNA sponge elements; in this way they post-transcriptionally provided mRNA escape miRNA repression [9, 10]. Besides, they are involved in cell cycle and other physiological cell processes by making complexes with proteins [11]. In recent years, they are attracting considerable research attribute to their roles in initiation and progression of human malignancies, especially cancers.

Ovarian cancer is one of the most common gynecologic disease worldwide. In 2018, more than 295,000 new cases were diagnosed and approximately 184,000 deaths were reported worldwide [12]. Despite develops in chemotherapy and surgical treatment, the survival rate is not favorable, because of poor prognosis and cancer recurrence [13]. Without doubt, clearing the circRNAs function and roles will improve knowledge of ovarian cancer and provide new chances and opportunities to develop more effective treatment or diagnostic approaches. Herein, we reviewed properties and function of circRNAs in cancers. We also highlight the circRNAs dysregulation and functions in the regulation of ovarian cancers.

### CircRNA biogenesis

In eukaryotic genes, non-coding introns that are between exons are removed after transcription by alternative splicing of pre-mRNA transcript. (in alternative splicing process of eukaryotic pre-mRNA, the introns that are non-coding region located between exons are removed after transcription.) [14]. After that, exons are reattached to each other and the spliced out introns can be formed linear or lariat molecules. Many evidence have demonstrated that circular RNAs are originated during splicing pre-mRNAs. like mRNAs, circRNAs are transcribed by RNA polymerase II (Pol II) but they have not terminal structure such as 3′ poly-A tail and 5′ Cap that exist in the fate of RNA transcripts [4]. The lack of free end in circRNAs lead to be resistant toward endonuclease and subsequently, are more stable than their linear counterpart.

**Fig. 1:**
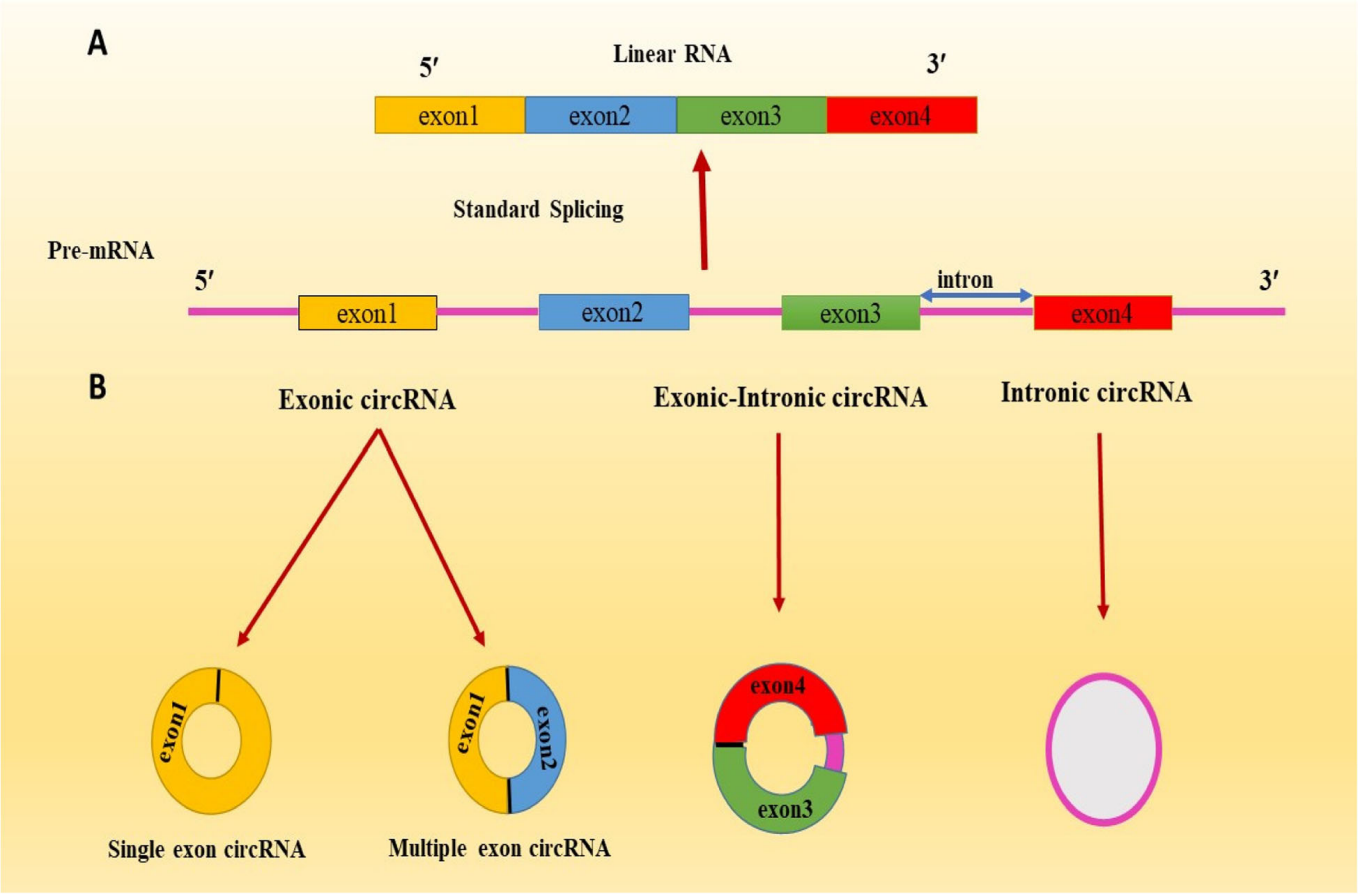
The kind of biogenesis of circRNAs: (**a**) Standard splicing (conventional linear splicing); (**b**) circRNAs is generated by a process called, Backsplicing: this splicing lead to creating 1) exonic circRNA that can make two forms including single exon circRNA and multiple exon circRNA, 2) Exonic-Intronic circRNA and 3) Intronic circRNA

Based on sequences and genomic origins, circRNAs are divided into three classes: intronic, exon-intron and exonic, which is the most prevalent form of circRNAs (Fig. 1) [15]. Exonic circRNAs are formed during back-splicing or Exon skipping and are usually catalyzed by the spliceosomal machinery [16]. In back-splicing, which also called circle splicing or head-to-tail splicing, the segments of spliceosome lead to attach the downstream splice donor (5′ end of the intron) to an upstream splice acceptor (3′end of the intron) and circular inverting RNA are produced by covalently linked ends. In multiple exons, a downstream end of exon splices to the initiation site of an upstream exon. It may also happen with single exon; in this case, the 3′end of one exon binds to the5′ end site [17, 18].

Exon skipping is the other model of splicing for producing of exonic circRNAs. In this process, when one or maybe more of the exons skipped, the introns with a loop structure, known as lariat (which resembles lasso), can formed exon-containing lariat during internal splicing [19–21].

Generally, in eukaryotic genes, introns undergoing standard splicing is separated and degraded by enzymes. However, some of the introns have conservative sequences at both ends of them. These motifs are GU-rich element (7-nucleotide) located at the near 5′ splice site and the C-rich element (11-nucleotide) at 3′-branch site. Due to preserve motifs, The lariat introns escape of debranching enzymes, consequently, RNAs can be circularized and circular intronic RNAs (ciRNAs) are produced [18, 22]. A feature of the intronic circRNAs that distinguishes them from exonic circRNAs is single unique 2′–5′ linkage, while in exonic circRNAs the phospholipid linkage is 3′-5′. CircRNAs are settled in the nucleus and have a role in the regulation of parent genes [23].

Finally, exon-intron circular RNAs (EIciRNAs) can be produced from circularized exons with intron preserved between exons (Fig. 1). According further studies, it is confirmed that circRNAs and EIciRNAs are involved in Pol II transcription and both of them play role in regulation of parental gene expression. Like ciRNAs, EIciRNAs are located in nucleus [17, 24].

Recently, it is shown that some classes of the enzyme are effective in circRNAs biogenesis. RNA binding proteins (RBPs) is a group of enzymes that have a role in the generation of circRNAs in some situations and is demonstrated to act as a regulator in activation or repression circRNAs. Actually, it is proven that they play a crucial role in the control of RNA such as splicing [25]. Ashwal-Fluss et al. are exposed in their research that RBPs can monitor muscle-blind protein (MBL) levels in the fly brain [26]. In this process, when MBL level is too high, RBPs attach to pre-mRNAs and prevent to convert linear RNA and cause circRNAs, afterward, translation stops and MBL protein has not produced [27]. ADAR (adenosine deaminases acting on RNA) is another enzyme that involved in circRNAs biogenesis and can regulate RBPs level. Since ADAR has the function in RNA editing, is imperative for mammalian development. In kinds of research, it is expressed that ADAR causes decreasing formation of circRNAs through debilitating and editing RNA duplexes [7, 28].

### Circular RNAs and their roles in cancer pathogenesis

Cancer is one of very important health problems [29, 30]. It has been showed that a variety of internal (genetics and epigenetic factors) and external factors involved in cancer pathogenesis [29, 31]. Among epigenetic factors, non-coding RNAs have critical roles in the pathogenesis of many diseases such as cancer, cardiovascular diseases, and diabetes [32–35]. Considering the many challenges associated the effects of non-coding RNAs such as lncRNAs and miRNAs and their role in cancer, nowadays a new category of these non-coding RNAs, called circRNAs, have attracted considerable attention. Many evidence in recent years have confirmed that circRNAs play an important role in the onset and progression of various cancers including gastric cancer, esophageal cancer, ovarian cancer, bladder cancer, renal carcinoma, oral carcinoma, lung cancer, and hepatic carcinoma (Table 1) [42, 63–66]. According to development of techniques that were able to discover function of circRNAs, it is proven that they can affect on several pathways and aberrant expression of them causes various disease including cancer. Recently, it is demonstrated that circRNAs in numerous cancers changes expression level or even can interact with microRNAs and act as sponge miRNAs.

**Table 1:** Selected circular RNAs in different cancers

Cancer	Circular RNA	Regulation of the expression in cancer	Genetic target (s)	Effect (s)	Model used	Sample type	Reference
GC	CiRS-7	Up	miR-7	Increase the overall survival	In vitro	Tissue, cell line	
	Circ_104,916	Down	Unknown	Promote TNM and lymph node metastasis	In vitro	Tissue, cell line	[36]
	Circ_0000181	Down	Unknown	Lymphatic metastasis and distal metastasis	In vitro	Tissues, plasma	[37]
BLC	CircHIPK3	Down	miR-558	Promote Vascular invasion and lymph node metastasis	In vitro*,* In vivo	Tissue, Cell line mice	[38]
	CircTCF25	Up	miR-103a miR-107 CDK6	Promote cell proliferation, Migration	In vitro	Tissue, Cell line	[39]
	CircRNA BCRC4	Down	miR-101	Association with cell apoptosis	In vitro	Tissue, Cell line	[40]
ESCC	Circ-ITCH	Down	miR-7 miR-17 miR-214	Inhibit the Wnt/β-catenin pathway	In vitro*,* In vivo	Tissue, Cell line	[41]
	hsa_circ_0067934	Up	Unknown	Promote the proliferation and migration	In vitro	Tissue, cell line	[42]
	circ-DLG 1	Up	miR-515 miR-942 miR-589 miR-136	Promote the esophageal cell proliferation ability	In vitro	Tissue, cell line, Plasma	[43]
BC	hsa_circ_0001982	Up	miR-143	Promote the proliferation and invasion and suppress apoptosis	In vitro	Cell line, Tissue	[44]
	CircMYO9B	Up	miR-4316	Promote the BC cell proliferation, migration and invasion, upregulation of FOXP4	In vitro*,* In vivo	Tissue, Cell line	[45]
	hsa_circ_0007534	Up	miR-593	Promote the BC cell proliferation, colony formation, and invasion, upregulation of Mucin 19 (MUC19)	In vitro	Tissue, Cell line	[46]
CRC	CircITGA7	Down	miR-370-3p	Promote the proliferation and metastasis in CRC cells via Ras pathway	In vitro*,* In vivo	Tissues, cell line	[47]
	hsa_circ_0000523	Down	miR-31	Promote the proliferation and suppressed apoptosis of CRC cells via indirectly regulating Wnt/β-catenin signaling pathway	In vitro*,* In vivo	Tissues, cell line	[48]
	Circ-001569	Up	miR-145	Promote the proliferation, cell growth and invasion of CRC	In vitro	Tissues, cell line	
HCC	CSMARCA5	Down	miR-17-3p miR-181b-5p	Promote the growth and metastasis of HCC	In vitro*,* In vivo	Tissues, cell line	[49]
	CircMTO1	Down	miR-9	Promote the HCC progression and cell growth via miR9-P21	In vitro*,* In vivo	Tissues, cell line	
	Circ_100,338	Up	miR-141-3p	Promote the lung metastasis, vascular invasion, and TNM	In vitro*,* In vivo	Tissues, cell line	[50]
OSCC	CircRNA_100290	Up	miR-29b	Suppress G1/S arrest, induce cell proliferation and upregulate the expression of CDK6	In vitro*,* In vivo	Tissues, cell line	[51]
	hsa_circ_0055538	Down	p53/Bax/Apaf1/ caspase-3/ p21/Bcl2	Development of OSCC via the p53/Bcl-2/caspase signaling pathway.	In vitro*,* In vivo	Tissues, Cell line	[52]
	CircDOCK1	Up	miR-196a-5p	Inhibit the cell apoptosis via miR-196a-5p/BIRC3 pathway	In vitro*,* In vivo	Tissues, Cell line	[53]
PCa	Circ-SMARCA5	Up	Unknown	Promote the cell cycle and inhibited cell apoptosis	In vitro	Cell line	[54]
	Circ-102,004	Up	ERK/JNK/ Hedgehog	Promote the cell proliferation and decrease cell apoptosis in PCa	In vitro*,* In vivo	Tissues, Cell line	[55]
	CircMYLK	Up	MiR-29a	Promote the PCa cell proliferation, invasion, and migration	In vitro	Tissue, Cell line	[56]
LC	Circ_100876	Up	Unknown	Promote the lymph node metastasis/TNM and overall survival	In vitro	Tissues	[57]
	CircFARSA	Up	miR-330-5p miR-326 FASN	Promote the cell migration and invasion	In vitro*,* In silico	Tissue, Plasma	[58]
	CircRNF13	Down	miR-93-5p	Inhibit the cell invasion and metastasis	In vitro	Tissues, Cell line	[59]
OS	hsa_circ_0001564	Up	miR-29c-3p	Promote the proliferation activity, repressed cell cycle arrest in G0/G1 phase, and suppressed apoptosis	In vitro	Tissues, Cell line	[60]
	circ-0016347	Up	miR-214	Promote the proliferation, invasion and metastasis	In vitro*,* In vivo	Tissues, Cell line	[61]
	circUBAP2	Up	miR-143	Promote the osteosarcoma growth and inhibit the apoptosis, and promote the osteosarcoma progression	In vitro*,* In vivo	Tissues, Cell line	[62]

*BC* Breast Cancer, *BLC* Bladder cancer, *CRC* Colorectal Cancer, *GC* Gastric Cancer, *ESCC* Esophageal squamous cell carcinoma, *LC* Lung Cancer, *OSCC Oral squamous cell carcinoma*, *HCC* Hepatocellular carcinoma, *OS* Osteosarcoma, *PCa* Prostate cancer, *TNM* tumor-node-metastasis, *EMT* epithelial-mesenchymal transition

During a study, the expression of circ-104,916 was investigated in gastric cancer (GC) by microarray. In this inquiry, circ-104,916 was downregulated in tissue GC compare with normal tissue. After that, they performed quantitative real-time PCR (in 70 pairs of human GC specimens) to confirm the results of the microarray and figured out that the results were matched. They also showed that the overexpression of circ-104,916 via pcDNA-circ-104,916 in two GC cell line (BGC823, MGC803) lead to significantly suppressed proliferation, invasion, and migration via changing the epithelial-mesenchymal transition (EMT) manner. According to the results, they claim that circ-104,916 would be appropriate biomarker for GC [36]. Recently, a novel circRNAs named Cdr1, antisense to the cerebellar degeneration-related protein 1 transcript, has been discovered. This circRNAs also called ciRS-7 which was because of the appearance as a powerful sponge for miR-7 [67, 68]. Since the result of qRT-PCR in a research demonstrated that ciRS-7 was overexpressed in GC samples compared with control tissues, it was suggested that this circRNAs could serve as a potential biomarker in GC. On the other hand, in this study the correlation between miR-7 and ciRS-7 was checked out. It was observed that the expression level of miR-7 was reduced in GC cell lines (MGC-803, HGC-27, [69]). MiR-7 is known as a tumor suppressor and can induce apoptosis in GC cell lines via modulation of the PTEN/PI3K/AKT pathway [70]. It is worth noting that overexpression of ciRS-7 has not straight effect on GC cell migration and proliferation but since they can act miR-7 sponge, lead to intensifying PTEN/PI3K/AKT pathway through repression of miR-7 [69].

The bladder cancer-related circular RNA-2 (BCRC-2), also known as circHIPK3, expression level was explored in bladder tissue. On the basis of their result, it was shown that circHIPK3 was markedly decreased in bladder cancer cell lines and tissues. Actually, the circHIPK3 suppressed the expression of heparanase (HPSE) through sponging miR-558 [38]. HPSE is a vital mammalian enzyme that able to destroy heparin sulfate polymer and has the ability in both the extracellular matrix and cell-surface. This enzyme has a critical purpose in preliminaries tumorigenesis, invasion, and angiogenesis many cancers [71]. Due to circHIPK3 functions as a tumor suppressor and miR-558 sponger in bladder cancer tissue, reinforced expression of circHIPK3 could suppress lymph node metastasis, angiogenesis, and vascular invasion in bladder cancer cell line and inhibit bladder cancer metastasis and growth, in vivo [38].

In 2015, a study has surveyed the alteration in the expression level of circ-ITCH in esophageal squamous cell carcinoma (ESCC) tissues. They have manifested that circ-ITCH could act as a tumor suppressor in ESCC and an increase in circ-ITCH expression ITCH leaded to represses the Wnt/β-catenin pathway through the destruction of phosphorylated Dvl2 via sponge of miR-7, miR-17 and miR-214. Their results also revealed an association between circRNAs, mRNAs, and protein and were proved that aberrant alteration among them may cause cancer beginning and progression. They have announced the down-regulation expression of cir-ITCH in ESCC tissue compare with matched normal tissue. Subsequently, the level of ITCH positively with circ-ITCH reduced and the expression of oncogenes including miR-7, miR-17 and miR-214 overexpressed in ESCC and the Wnt/β-catenin pathway not be repressed [41].

A significant overexpression of circMYO9B was reported in breast cancer (BC) tissue. Specific siRNA against circMYO9B caused suppression in proliferation, invasion and migration in MCF7 and MDA-231 cell lines. Likewise, the in vivo assay had shown a decrease in level circMYO9B tumor growth. They also described the mechanism of circMYO9B that sponged miR-4316 and enhanced expression of FOXP4 which act as tumor suppressors and oncogene, respectively [72, 73]. Clearly, the expression of miR-4316 was negatively related to circMYO9B and FOXP4 in BC tissues. Finally, they introduced circMYO9B as a prognostic biomarker for the BC patients and announced that CircMYO9B/miR-4316/FOXP4 axis performed a crucial purpose in BC pathogenesis [45].

Another group of scientists had reported a novel circRNA named circITGA7 which was markedly downregulated in colorectal cancer CRC) tissue and cell lines. They also found that the linear form of ITGA7 has been downregulated in this cancer. The downregulation of circITGA7 and ITGA7 were associated with CRC progression. To the examine circITGA7 and ITGA7 roles in CRC, ectopic circITGA7 were overexpressed and observed proliferation and metastasis repression in CRC cells, in vitro and in vivo. CircITGA7 could sponge miR-370-3p and indirectly leaded to an increase in neurofibromin 1 (NF1) which is its target and a negative regulator of Ras pathway. On the other hand, CircITGA7 through suppressing RREB1, a transcription factor which activate Ras pathway transcription factor, leaded to ITGA7 overexpression. RNA-Seq and KEGG pathway analysis showed that ITGA7 may be concomitant in cytokine-related signaling pathways [47].

It is well-known that the miR-17-3p and miR-181b-5p overexpression are contribute to the progression of hepatocellular carcinoma (HCC) [74, 75]. Yu et al. have shown that a circRNA named cSMARCA5, might regulate expression of miR-17-3p and miR-181b-5p oncomirs. But, they also have identified that cSMARCA5 is downregulated in HCC tissue and cells while mRNA and protein form of cSMARCA5 that is SMARCA5 were overexpressed. It has been noted that cSMARCA5 via sponging miR-17-3p and miR-181b-5p can promote expression of a tumor suppressor called TIMP3. Likewise, they determined that the downregulation of cSMARCA5 is an association of a tumor-promoter gene DHX9 in HCC. On the basis of results, it is concluded that cSMARCA5 may prevent the growth and metastasis of HCC and work as a prognostic biomarker in HCC patients [49].

One report described the expression and function of Circular SMARCA5 (circ-SMARCA5) in prostate cancer (PCa). Due to circ-SMARCA5 up-regulation in PCa tissues and cell lines, it is known as oncogenic circRNAs in this cancer. The double hydrogen testosterone (DHT), is a natural hormone and can be used as a medication, treatment resulted in circ-SMARCA5 down-regulation which can be concluded that it is an androgen-inducible circRNA. The circ-SMARCA5 silencing in DU145, a PCa originated cell line, inhibited cell cycle, cell proliferation and motivated apoptosis in PCa cells [54].

The circFARSA, derived from phenylalanyl-tRNA synthetase alpha chain (FARSA) gene, has been upregulated in non-small cell lung cancer (NSCLC) tissue compared to non-cancerous tissue. The circFARSA overexpression leaded to migration and invasion NSCLC cells. In silico analysis and HITS-CLIP experiment showed that miR-330-5p and miR-326 counterparted in the fatty acid synthesis and they were introduced as circFARSA direct targets. It is the hypothesis that circFARSA act as the sponger for miR-330-5p and miR-326 and subsequently when overexpressed lead to upregulation of FASN, a target of both miRNAs [57].

CircNOL10 can be described as a round RNA whose expression takes place in low amounts when experiencing lung cancer, although there is no clear information on the functioning mechanisms in this disease [45]. Nan and colleagues evaluated the role and molecular processes of circNOL10 in their study of lung cancer advancement in the laboratory conditions as well as in living tissues [45]. According to their findings, circNOL10 could considerably hamper progress of lung cancer, while regulation of its expression was concurrent with methylation of its parental gene named Pre-NOL10 as well as intertwining factor of epithelial splicing regulatory protein 1 or ESRP1.

CircNOL10 elevates the expression of the transcribing factor of sex comb on midleg-like 1 (SCML1) through inhibition of ubiquitination, which subsequently influences the balance of HN polypeptide group through SCML1. Moreover, circNOL10 can influence the performance of mitochondria by regulation of the HN polypeptide group along with its effects on several signaling routes, which will eventually hamper multiplication of the cell along with the development of its cycle. As a result, apoptosis is promoted in the lung cancer cells and results in the inhibition of their growth.

The present research aimed to find out how circNOL10 acts and what molecular mechanisms are involved in advancement of lung cancer in addition to indicating its engagement in the transcriptional regulation of the HN polypeptide group via SCML1. According to the findings, HN could hamper the development of lung cancer cells. The information obtained through this study may be helpful in identification of new molecular treatments for lung cancer [45].

In oral squamous cell carcinomas (OSCC), circRNA_100290 was significantly upregulated in tissue compared with adjacent non-cancerous tissue. Actually, it was represented that circRNA_100290 regulate CDK6 expression with the competing endogenous RNA (ceRNA) mechanism. CircRNA_100290 could act as spongers for miR-29 family such as miR-29a, miR-29b, and miR-29c. Among them, it was clarified that CDK6 is a direct target of miR-29b. On the basis of considerations, it was exposed to circRNA_100290 and CDK6 were up-regulated in OSCC. To investigate the circRNA_100290 function in OSCC cell lines, the CDK6 expression was downregulated and caused cell proliferation in vitro and in vivo with knocking down of this circRNA [51].

The last circular RNA that introduced in this part is circ_001564. The circ_001564 was highly upregulated in osteosarcoma tissue and cell lines. In order to represent the circ_001564 mechanisms, it was knocked down in osteosarcoma cell lines (HOS and MG-63). The results manifested restraining the cell proliferation, induced cell cycle arrest in G0/G1 phase and increased apoptosis in osteosarcoma cell lines. Base on bioinformatics analysis, miR-29c-3p might be bonded to circ_001564 complementary; consequently, the oncogenic effect of hsa_circ_001564 was be reversed. In addition, the circ_001564 had function as miR-29c-3p sponge that proposed competing endogenous RNAs (ceRNAs) mechanisms in osteosarcoma [60].

### CircRNAs in exosomes

For the first time, Huang et al., considered the existence of circRNAs in exosomes [55]. As a result of this finding new horizons were provided to examine circRNAs. In total, locating circRNAs in the cytoplasm can be done more conveniently, while different sorts of circRNAs can be found in exosomes of various kinds of cytoplasm, which in turn indicates the potential active regulation of special circRNAs transfer to exosomes through a selection-based procedure. Nevertheless, there is no clear information available on the mechanisms through which transfer of circRNAs into exosomes takes place.

Two presumptions can be considered regarding how circRNAs act in exosomes. According to the first one, they facilitate cell correspondence through the existing substance in exosomes, making distal cells’ material and transmission of information possible. Based on the second assumption, circRNAs in exosomes assist in elimination of circRNAs collected in cells. However, recent studies do not provide clear information on these presumptions. Lasda et al. endeavored to examine this assumption and they accordingly hypothesized that circRNAs in exosomes along with extracellular vesicles could play the role of a mechanism removing endogenous circRNAs [56].

Analysis of the corresponding number of circRNAs along with linear RNAs was performed in the present study taking extracellular vesicles (consisting of exosomes) of the Hela, 293 t, U2OS cells into account. According to the findings circRNAs outperformed the linear RNAs. Accordingly, it was stated that cells would probably have the potential to manage circRNAs by their excretion through extracellular vesicles. Since, it would be possible fot the EVs to be captured by other cells, such as macrophages, their function as message carriers could be possible in order to maintain intercellular communication. This is a new presumption which of course needs to be validated.

Enrichment of circRNAs was performed in another study in platelet due to exonuclease [58]. But the question was whether similar causes and findings could be found when linear RNA was broken down. Consequently, this potential issue was not thoroughly disapproved in the present paper. Down-regulation of circRNAs takes place in the colon of mutant KRAS, while its secretion could be accomplished in the exosomes [59].

Three types of colorectal cancer cell lines were investigated in the present research, including DLD-1(wild-type along with G13D abnormal KRAS alleles), DKO-1 (just abnormal KRAS allele) as well as DKs-8 (just wild-type KRAS allele). The circRNAs of the three mentioned cell types underwent analysis through RNA-Seq. Considerable down-regulation of circRNAs was observed in DLD-1 as well as DKO-1 cells rather than DKs-8 cells.

Later, examination of two other colorectal cancer cell lines including HCT116 (abnormal KRAS allele) and HKe3 (the kind of wild KRAS allele) went on, with the findings obtained from sequencing confirming the fact that overall expression of circRNAs was higher in HKe3 compared with HCT116. CircRNAs associated with colorectal cancer is found in exosomes and considerable down-regulation of circFAT1 was observed contrary to up-regulation of circRTN4. However, there were no evident changes in the respective linear mRNA which was under regulating effects of these genes as it was evident in circRNAs. Moreover, it could be stated that transmission of cirRNAs into exosomes would be performed through an elective procedure [59].

### Circular RNAs and ovarian cancer

Accumulating studies have confirmed interplay between circRNAs and human disorders including embryonic development, Alzheimer’s disease, diabetes and also cancers [15, 76]. Ovarian cancer is one of the major prevalent and causing cancer mortality in female [77]. Dysregulation of signaling pathways and cellular mechanisms have crucial role in cancer initiation, progression and invasion [69, 78]. Regarding regulatory roles of circRNAs and ovarian cancer impotency, the correlation and relation between them are attractive for researchers. In recent years, numerous studies focused on differentially expressed circRNAs and their function in ovarian cancers, suggesting circRNAs as potentially novel biomarkers or therapeutic agents in this disease (Table 2).

**Table 2:** Studies concerning Circular RNAs and Ovarian Cancer

Circular RNA	Regulation of the expression in cancer	Genetic target (s)	Effect (s)	Model used	Type of cell line/ Number of sample (Human)	Reference
circ_0031356, 0093477, 0110166, 0126526, 0130590 and 0135175)	Down regulated	–	–	In silico	–	[79]
Circ-ITCH	Down regulated	miR-145	Up regulation of RASA1, inhibit the tumorigenesis	In vitro*,* In vivo and Human	SK-OV-3 and Caov-3 cell lines, BALB/c nude mice/20	[80]
circ_0004712, circ_0002198, circ_0008951, circ_0017248 and circ_0003570	Up regulated Up regulated Up regulated Up regulated Down regulated		Upregulation of SCN3B, ENTPD1, BACH2, C3 and G0S2, down regulation of CKS2 and PGRMC1	Human	41	[81]
VPS13C-has-circ-001567, RAD50-has-circ-00718, and SPECC1-has-circ-000013	Up regulated		Increase the proliferation, migration, invasion E-cadherin and N-cadherin	In vitro*,* In vivo*,* Human	SK-OV-3, OV-1063 cell lines/20	[82]
circEPST1	Up regulated	miR-942	Upregulation of EPST1	In vivo*,* In vitro	OV119 and A2780 cell line/50	[83]
Circ-CSSP1	Up regulated	miR-1236-3p	Upregulation of ZEB1, and promote the EMT transition	In vitro*,* In vivo	SK-OV-3, A2780 and CAOV3 cell lines	[84]
Circ-HIPK3	Up regulated	–	Promote the lymph node invasion and advanced stage	In vitro, Human	,A2780, HO8910, SK-OV-3 and CAOV3 cell lines/69	[85]
Circ-HIPK3, circRHOBTB3, circPCMTD1, circSETD3, circATRNL1, circAC139769.1	Down regulated Down regulated Down regulated Down regulated Down regulated Up regulated		Promote the apoptosis and inhibit the proliferation and invasion	In vitro, Human	A2780,SK-OV-3,HO8910, OVCAR8,COC1and IOSE80 cell lines /21	[86]
circHIPK3, circRNA2058, circRNA3336, circRNA2606, circRNA1656, circRNA1312 and circRNA7474	Down regulated Down regulated Down regulated Down regulated Down regulated Up regulated Up regulated	–	–	In vitro, Human	SK-OV-3, HO 8910, OVCAR-3, and A2780 cell lines/60	[87]
hsa_circ_0051240	Up regulated	miR-637	Upregulation of KLK4	In vitro*,* In vivo, Human	CAOV-3, SKOV-3, OVCAR-3 and H8910/ 33	[88]
Circ-ITCH	Down regulated	miR-10a	Enhance the apoptosis	In vitro	SKOV3, A-2780, OVCAR-3 and HO-8910	[89]
Circ-LARP4	Down regulated		Lower expression is associated with advanced grade	Human	78	[90]
circBNC2, circEXOC6B, circFAM13B, circN4BP2L2, circRHOBTB3 and circCELSR1	Down regulated, Down regulated, Down regulated, Down regulated, Down regulated, Up regulated			Human	54	[91]

*BACH2* BTB Domain And CNC Homolog 2, *CKS2* Cyclin-dependent kinase subunit 2*, EMT* epithelial–mesenchymal transition, *EPST1* epithelial-stromal interaction 1, *G0S2* G0/G1 switch gene 2, KLK4:Kallikrein-related peptidase 4, *RASA1* RAS P21 Protein Activator 1, SCN3B Sodium channel subunit beta-3, *PGRMC1* Progesterone receptor membrane component 1, *ENTPD1* Ectonucleoside Triphosphate Diphosphohydrolase 1, ZEB1 Zinc finger E-box-binding homeobox 1

For the first time, Ahmed, et al., performed a robust circRNAs expression analysis in clinical ovarian tumors. They reported that abundant number of circRNAs differentially expressed in tumor samples. They also examined differential mRNA and circRNA expression signatures between primary and metastasis ovarian tumor. Regarding the RNA exonuclease resistance and stability of circRNAs [92], they distinguished suitably the primary tumor from metastatic lesion rather mRNAs. Interestingly, signaling pathways such as STAT, AKT, NF-kB, TGF-B, ILK and HGF and VEGF that are typically activated for linear RNA producing and angiogenesis are downregulated for circRNA in ovarian metastasis cancer [93]. Also, miR-24 and Let-7, well-known tumor suppressor miRNAs [94, 95], expressed in low level in primary tumor. The high expressed candidate circRNAs encompass several binding sites, suggesting the possible sponge roles of them for both miRNAs. Over all, the circRNAs/ miRNAs/ mRNAs cross talk indicated potentially role of circRNAs as tumor activator of inhibitor [93].

The circ-CSPP1 or circ-0001806 is derived from centrosome/spindle pole-associated protein 1 (CSPP1) gene which is located in 8q13.2 region of human genome. The oncogenic function of CSPP1 have determined in luminal breast cancer and B-cell lymphoma [96, 97]. Also, the expression level of circ-CSPP1 was performed in border line, benign, ovarian tumor tissues, and non-tumor tissues. The circ-CSPP1 expressed remarkably high in borderline and tumor tissues rather benign and cancer tissues. Consistently, this circRNA expression was correlated with stage II-VI. Subsequent knocking down of circ-CSPP1 caused an arrest in cell growth, invasion and migration and inversely, the overexpression stimulated cancer properties. A negative correlation between miR-1236-3p and circ-CSPP1 was observed in ovarian cell lines. Dual luciferase assay revealed that circ-CSSP1 acted as miR-1236-3p decoy [84]. Reportedly, miR-1236-3p suppressed tumor development through targeting zinc finger E-box binding homobox 1 (ZEB1) in ovarian cancer [98]. Thus, circ-CSPP1 sponged miR-1236-3p and attenuated its silencing effect on ZEB1, consequently stimulated EMT and cancer development. Moreover, the expression of vascular endothelial growth factor A (VEGFA) and matrix metalloproteinase-2 (MMP2), which are oncogenic proteins, decreased significantly in silenced circ-CSPP1 cell line [84].

The overexpression of circRNA-000479, or circEPSTI1 was reported in triple-negative breast (TNBC) cancer tissues. The high level of circEPSTI1 was correlated with reduced survival in these patients. The circEPSTI1 promoted proliferation and clonal formation and suppressed apoptosis in TNBC cell lines. Moreover, xenograft and invitro experiments confirmed its role as a decoy of miR-4753 and miR-6809 to modulate BCLI 1A [99]. The circEPSTI1 also played an important role in ovarian cancer regulation. Compared to adjacent non-tumor tissue, the circEPST1 was overexpressed in 50 paired ovarian cancer sample. Also, the circEPSTI1 silencing impaired cell growth, invasion but induced apoptosis. Knocking down of circEPSTI1 leaded to a decrease in ovarian tumor size and lung metastasis in mouse xenograft which indicated the circEPSTI1 role in ovarian cancer progression and metastasis invivo. Based on experimental assays, this circRNA mostly localized in cytoplasm was capable to sponge miR-942, which was down regulated in ovarian cancer tissues. EPSTI1, linear form of circEPSTI1, also overexpressed in ovarian cancer tissues. Luciferase assay showed that miR-942 directly targeted EPSTI1, indicating the circEPSTI1 effect on EPSTI1 [83].

The role of circHIPK3 was investigated in some human malignancies. For instance, the circHIPK3 overexpression was reported in prostate cancer tissues which was correlated with tumor stage. This circRNA relieved MCL1 expression by sponge miR-193-3p [100]. Its expression level and function was tested and the data revealed upregulation of circHIPK3 in lung cancer cells compared with lung epithelial cells. It could promote oncogenic properties by acting as a decoy for miR-124 and subsequently causing upregulation in its target including SphK1, CDK4 and STAT3 [101]. This circRNA could sponge multiple miRNAs including miR-124, miR-584, miR-29a, miR-29b, miR-379 and miR-558 [38, 102].

Also, it was investigated the circHIPK3 expression correlation with clinicopathological factors in the tissues associated with ovarian cancer.

The circHIPK3 profiling revealed an increased expression in tumor samples. The over expressed cases suffered from node invasion and inappropriate prognosis [85]. Contradictory, it has been reported that circHIPK3 expressed in low level in epithelial ovarian cancer, but it was highly expressed in IOSE80 cell, a normal ovarian tissue cell line. In addition, the authors claimed that knocking down of circHIPK3 induced cell growth, migration and metastasis and repressed apoptosis of ovarian cancer cells [86]. The circ-0051240 is another circRNA that play a crucial role in ovarian cancer. The over expression of circ-0051240 in ovarian cancer tissues enhanced tumor formation, cell growth, progression and invasion, invivo and invitro. It was identified that the circ-0051240 affected via tapping miR-637 which post-transitionally inhibited Kallikrein 4 (KLK4) expression [88]. It was reported that the KLK4 overexpression was related with more aggressive and poor-prognostic ovarian cancer [103].

Emerging evidence represented the over expression and oncogenic role of miR-10a in ovarian cancer [104, 105]. Luo and colleagues indicated a reduction in circ-ITCH expression in cancerous ovarian cell lines in comparison with non-cancerous ones and its effect on miR-10a level. The over expression of circ-ITCH promoted apoptosis and inhibited cell proliferation in ovarian cancer cells. The miR-10a expression was adversely regulated with circ-ITCH and the miRNA overexpression compensated the circ-ITCH inhibition effects on cell growth. Interestingly, the circ-ITCH expression was independent with miR-10a expression [89]. The circ-LARP4 is another down regulated circRNA in ovarian cancer tissue. The low expression of circ-LARP4 was considerably associated with lymph node invasion and also poor prognosis. Hence, it might be used as a potentially novel biomarker for ovarian cancer detection [90].

## Conclusion

Previously, circRNAs were known as errors during RNA splicing. Developing technology in recent years improved our knowledge about this class of lncRNAs. Nowadays, they are understood to be a frequently stable RNA molecule which expressed in various tissues and cells. The circRNAs represent a miRNAs decoy role to indirectly modulate their target genes. Accumulating evidences revealed the critical regulatory roles of them in tumorigenesis, with cancer-type specific distinguishable expression levels. CircRNAs biological functions, such as interactions with specific mRNA, protein and specifically miRNAs provide a regulatory network for cancer progression and invasion, so promise to providing more reliable, sensitive diagnoses and more effective cancer therapy. Based on reports, ovarian cancer is the sixth and one of the lethality cancer type in women worldwide. Without doubt, accurate knowledge about regulating molecular mechanisms, including circRNAs role provides a promising opportunity to better understanding of ovarian cancer. Subsequently novel approaches can be identified to early diagnosis, prognosis, follow up and therapeutics of ovarian cancers.

## Data Availability

The primary data for this study is available from the authors on direct request.
